# A feasibility study: a non-inferiority study comparing head-mounted and console-based virtual reality for robotic surgery training

**DOI:** 10.3389/frobt.2025.1616462

**Published:** 2026-01-06

**Authors:** Kazuho Kawashima, Shadi Ghali, Justin W. Collins, Ali Esmaeili

**Affiliations:** 1 Division of Surgery & Interventional Science, University College London, London, United Kingdom; 2 Department of Plastic and Reconstructive Surgery, Royal Free NHS Foundation Trust, London, United Kingdom; 3 Consultant Plastic Surgeon, London, United Kingdom; 4 University College London, London, United Kingdom; 5 Division of Uro-Oncology, University College London Hospital, London, United Kingdom; 6 Executive Medical Leadership, Bayes Business School, London, United Kingdom

**Keywords:** virtual reality, robotic assisted surgery, surgical education, technology, Versius

## Abstract

**Background:**

Head-mounted virtual reality (VR) simulations are increasingly explored in healthcare, particularly in patient education, stroke rehabilitation, and surgical training. While VR-based simulation plays a growing role in robotic-assisted surgery (RAS) training, the implications of head-mounted VR in this context remain underexamined.

**Method:**

This prospective, randomised, controlled trial with a single-arm crossover compared two training modalities: a head-mounted VR simulation and a conventional console-based simulation. Participants in the experimental group used head-mounted VR as their primary training method, while the control group trained on a conventional console. Both groups completed a running suture task at baseline, midterm, and final assessments on the surgical console. The primary outcome was the composite score from the final assessment.

**Results:**

Fourteen participants were equally distributed between the two arms. Baseline scores showed no significant differences. A two-way repeated measures ANOVA demonstrated significant overall improvement across assessments (F (1.688, 20.26) = 48.34, p < 0.001, partial η^2^ = 0.80). No statistical difference was found in final composite scores (mean difference: 8.4 ± 9.45, p = 0.391, Cohen’s d = −0.48), midterm scores, or granular kinematic data. However, non-inferiority could not be established as the confidence interval fell outside our pre-set margin. The crossover group required less time (mean difference: 39 ± 9.01 min, p = 0.004) and fewer attempts (mean difference: 8 ± 2.2, p = 0.009) to reach benchmark performance compared to the control group.

**Conclusion:**

Both head-mounted VR and console-based training significantly improved fundamental RAS skills in novices. While our study showed that the VR training shortened the time and attempts required to reach proficiency benchmarks, the small scale of this trial and the breadth of the confidence intervals mean the results should be viewed as preliminary observations. These results provide an initial signal of feasibility that warrants confirmation in larger studies.

## Introduction

Head-mounted virtual reality (VR) simulations are being rapidly explored in healthcare, with applications ranging from patient education and stroke rehabilitation to pain management (Tursø-Finnich et al., 2023; van der Kruk et al., 2022; Laver et al., 2017; Vassantachart et al., 2022; Kawashima et al., 2024). In the field of surgery, this technology has also been utilised for training surgeons in various procedures, such as laparoscopic surgery (Gurusamy et al., 2009).

Virtual simulations for robotic-assisted surgery (RAS) training were first described as early as 2006, but many of these systems, such as the da Vinci Surgical System (dVSS) (Intuitive), require access to costly robotic surgery consoles (Halvorsen et al., 2006). Although alternatives like the RobotiX Mentor (Surgical Science) eliminate the need for such consoles, the specific utility of head-mounted VR for robotic surgery training has received limited attention (Beulens et al., 2021; van der Leun et al., 2022).

Despite this, computer-based simulations have become an essential component in RAS training, allowing surgeons to build both basic and procedural skills with proven effectiveness (Kawashima et al., 2024; Gurusamy et al., 2009). This has led to many curricula incorporating virtual simulation as part of training programs for certifying surgeons (Khan Mustafa Tamim et al., 2023/06).

However, access to RAS training remains a significant challenge. A 2015 survey revealed that only 18% of general surgery residents in the United Kingdom had access to a robotic surgery console during their training (Farivar et al., 2015). Meanwhile, a 2024 study found that 72% of medical students at a single institution believed that a robotic surgery curriculum should be incorporated into medical education (Vigran et al., 2024/10). These findings highlight the growing interest in RAS among trainees and the critical need for more accessible and widespread opportunities to gain exposure and hands-on experience.

The barriers to implementing RAS training on a larger scale often stem from the financial and logistical challenges associated with purchasing and maintaining surgical consoles, as well as the physical space they require. In this context, head-mounted VR simulations offer a promising alternative. They represent a cost-effective and space-efficient approach that could make RAS training more accessible to medical students and novice surgeons.

This study explores the use of the Versius Trainer (CMR Surgical), a fundamental robotic skill (FRS) VR simulator used in a commercially available headset (VT-VR). We aimed to achieve two main objectives: first, to assess the feasibility of VT-VR training through a non-inferiority trial comparing VT-VR to the traditional surgical console training method, and second, to determine if VT-VR training could shorten the learning curve and reduce the time required on the surgical console to achieve benchmark performance.

## Methods

This study was designed as a prospective randomised controlled trial. All procedures adhered to relevant guidelines, and this article was prepared in accordance with the CONSORT statement and ICMJE recommendations.

### Materials

Two different devices were used for this study. The first is the Versius Trainer (VT), which is a robotic surgery console adapted into a virtual simulation. The second device is the VIVE Focus 3, which is a commercially available VR head-mounted display (HMD). A virtual simulation was installed onto the headset, turning the headset into Versius Trainer in Virtual Reality (VT-VR). Images of the devices can be seen in Supplementary Appendix 1.

### Participants

Medical students were recruited from schools within London between the 2nd and 16th of June 2024. The participants had to be novices with no prior experience in manipulating robotic surgery systems. Written consent was obtained from all participants prior to the start of the study.

### Tasks

The same virtual fundamental robotic skill (FRS) skill simulation was installed into VT and VT-VR. Of the 16 simulations available on the virtual simulation, 6 tasks were selected in increasing difficulty to teach participants FRS skills. The assessment was carried out using the most complex task on the simulation, which is the running suture. The specific learning points and images of each FRS task are outlined in Supplementary Appendix 2, 3.

### Study design

Participants were randomised into the VT-VR group (intervention) and the VT group (control). Both groups then underwent a baseline assessment on the VT using the running suture task. Both groups then participated in parallel training sessions until the final assessment. The six FRS tasks were distributed evenly over three sessions, each lasting 90 min. Midterm and final assessments were conducted after the second and third sessions, respectively, using the running suture simulation on the VT.

Participants in the intervention group were subsequently required to complete the same six FRS tasks on the VT (crossover group) to measure the time taken to complete all tasks. Although precautions were taken to mitigate carry-over effects by ensuring three to 4 days before crossing over, this study specifically aimed to measure the carry-over effects from VT-VR to VT by evaluating the time required to reach the benchmark for the FRS tasks.

### Endpoints

The primary endpoint of the study is the composite score calculated from the final assessment. The secondary endpoints included the composite score of the midterm assessment, kinematic data from baseline to final assessments, and the number of attempts, as well as the time required to reach predefined benchmarks for six FRS tasks using the VT system.

### Benchmarking and composite score calculation

For each FRS task, the participants were required to reach a benchmark score to progress to the following task. Each task has kinematic metrics with preset thresholds, and all thresholds must be met for the benchmark.

Scoring the participants based solely on the kinematic metrics had several limitations. The simulation only reports the scores in increments of 20 based on task accuracy/efficiency, and as there is increased type I error from the limited number of participants, the kinematic metrics are unable to detect subtle performance differences, reduce data granularity, and compromise statistical accuracy. To address this issue, a composite variable was derived from the overall score and time to completion using the min-max normalisation method (Song et al., 2013; Cinelli et al., 2021; Matteo Mazziotta, 2013). Both overall scores, including task accuracy and efficiency and time, are metrics which have previously been shown to reflect overall competency (Gurusamy et al., 2009; Cho et al., 2013).

The overall score was normalised using the following function:

$$Normalised\;Overall\;Score\;\left(NS\right)=\frac{S-S_{\min}}{S_{\max}-S_{\min}}$$

Where *S* is the observed overall score calculated by the simulation, and *S*_min_ *= 0* and *S*_max_ *= 100.* This formula scales the scores to a range of 0–1, where higher values indicate better performance.

The time to complete the assessment was normalised using the following function:

$$Normalised\;Time\;\left(NT\right)=1-\frac{T-T_{\min}}{T_{\max}-T_{\min}}$$

Where *T* represents the observed time, *T*_min_ *=* 180 s and *T*_max_ *= 1800 s.* Subtracting the fraction from 1 ensures that a shorter completion time results in a higher normalised value. *T*_max_ was determined based on the maximum allotted time for the assessment, while *T*_min_ was derived from trial runs conducted with an expert surgeon.

The final composite score is derived by integrating the normalised time (NT) and normalised overall score (NS) metrics through a weighted summation, with the result scaled to 100. The formula for the composite score is:

$$Composite\;Score=100\times \left(\left(\frac{5}{6}\times NS\right)+\left(\frac{1}{6}\times NT\right)\right)$$

The weight was evenly distributed between all metrics included in this composite score. This includes combined instrument tip path length, combined instrument angular path, needle dropped, number of failed stitch attempts, combined instruments out of view and time to completion.

## Statistics and non-inferiority margin

All data, except for the raw overall scores of the assessments, were normally distributed. For normally distributed data, including the calculated composite scores, comparisons between the control and intervention groups were performed using independent sample t-tests with Welch’s correction. To assess the overall treatment effect across all three assessments, a two-way repeated measures analysis of variance (ANOVA) was used. Cohen’s d and Partial eta squared (η^2^p) were used to calculate the effect size between the two groups and the overall treatment effect, respectively. All analyses were conducted using GraphPad Prism version 10.2.3 (GraphPad Software).

The non-inferiority margin was defined as 20 points, corresponding to the smallest possible scoring increment on the Versius Trainer platform. This threshold was selected based on prior validation work for the Versius system, which demonstrated that 20-point score differences are sufficient to discriminate between novice and expert performance levels (Althunian et al., 2017; Schumi and Wittes, 2011; Bjerrum et al., 2023). However, given the feasibility design and limited sample size, the selected margin should be regarded as pragmatic and provisional, with future work needed to refine non-inferiority thresholds using larger datasets or consensus methods.

### Sample size and randomisation

The sample size for this study was calculated based on the predetermined non-inferiority margin. With an anticipated standard deviation of five and a non-inferiority margin of ten, we estimated that a total of 12 participants would be required to achieve a statistical power of 95% and a two-sided alpha error of 5%. Participants were randomised into two groups following enrollment by generating a random number for each participant using the RAND function in Microsoft Excel.

## Results

**FIGURE 1:**
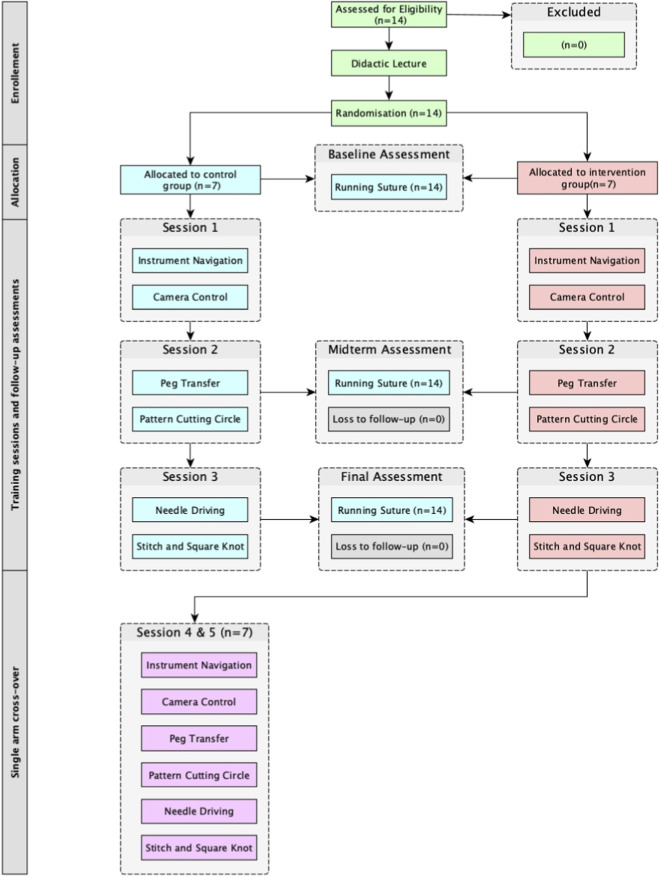
CONSORT flow diagram participant characteristics.

Following an initial eligibility assessment of 14 individuals, no participants were excluded. Consequently, 14 participants were enrolled, randomised, and assigned to either the intervention group (n = 7) or the control group (n = 7). All participants in both study arms completed the sessions and assessments. The CONSORT flow diagram is presented in Figure 1.

Demographic information for the participants was collected and tabulated. No statistically significant differences were observed between the two groups regarding composite scores, time, and all other recorded metrics. The complete results of the participant demographics are presented in Table 1.

**TABLE 1:** Participant demographics.

	Total (n = 14)	Intervention (n = 7)	Control (n = 7)
Age (years)	23.6 (3.3)	25 (4.4)	22.3 (0.5)
Gender			
Male	6	3	3
Female	8	4	4
Handedness			
Right	14	7	7
Left	0	0	0
Cases assisted in open surgery (n)	8.4 (10.9)	8 (10.6)	8.7 (12)
Cases assisted in laparoscopic surgery (n)	1 (1.6)	1 (1.3)	1 (1.9)
Cases assisted in robotic surgery (n)	0.1 (0.5)	0.3 (0.8)	0 (0)
Gaming hours/week	3.4 (3.3)	5 (3.6)	1.7 (2.1)
Plays instruments	6	3	3
Plays sports regularly	9	4	5

### Composite score

**FIGURE 2:**
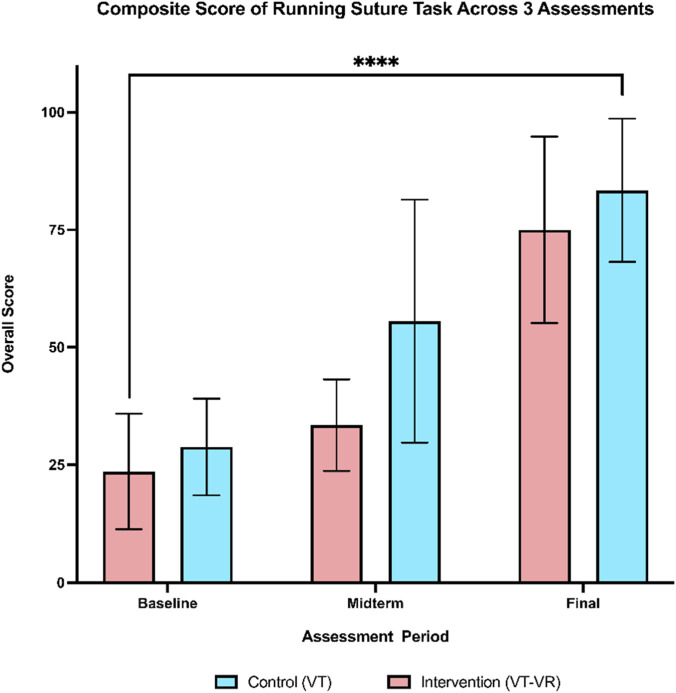
Composite score of running suture across 3 assessments. A comparable training effect can be seen by the final assessment. There is a statistically significant increase in the means of both groups from baseline to the final assessment. **** = <0.0001.

Participants in both the intervention and control groups improved their composite scores of running suture with a change score from baseline to the final assessment of 51.4 and 54.6, respectively. Furthermore, the two-way repeated measures ANOVA revealed a significant treatment effect over 3 assessment periods on overall scores (F (1.688, 20.26) = 48.34, p < 0.00, partial η^2^ = 0.80), indicating significant improvements from baseline to the final assessment for both groups (Figure 2 *).*

When comparing the overall scores of each assessment independently, there were no statistically significant differences between the two groups across all three assessments. Whilst it did not reach statistical significance, the control group showed a more rapid improvement at the midterm assessment with a large effect size (mean difference of 22.2 ± 10.4, p = 0.069, Cohen’s d = −1.14). However, by the final evaluation, the intervention group showed a level of improvement comparable to that of the control group (mean difference of 8.4 ± 9.45, p = 0.391, Cohen’s d = −0.48). The change score from baseline to final assessment also showed no statistical difference with a small effect size (Table 2). Regarding the non-inferiority, the upper bound of the confidence interval (29.0) fell beyond the pre-specified non-inferiority margin of 20, thus failing to reject the null hypothesis that training on VT-VR is non-inferior to console-based simulation training.

**TABLE 2:** Comparison of Composite Scores Across 3 Assessments. Change score is calculated by subtracting the final assessment score from the baseline assessment score. Individual P values were calculated using an independent t-test with Welch’s correction.

Composite scores of running suture (mean and SD)					
	Intervention (n = 7) mean (SD)	Control (n = 7) mean (SD)	Difference of means (95% CI)	P value	Cohen’s d
Baseline assessment	23.6 (12.3)	28.8 (10.3)	5.2 (−8.0–18.4)	0.406	−0.46
Midterm assessment	33.4 (9.7)	55.6 (25.8)	22.2 (−2.1–46.4)	0.069	−1.14
Final assessment	75.0 (19.8)	83.4 (15.2)	8.4 (−12.1–29.0)	0.391	−0.48
Change score	51.4 (16.0)	54.6 (15.43)	3.2 (−15.1–21.5)	0.710	−0.20

### Kinematic data: Midterm and final assessment

An analysis of the granular performance data from both the midterm and final assessments revealed no statistically significant differences between the two groups across all evaluated metrics. These metrics encompass time to task completion, efficiency scores, and error scores. A summary of all kinematic data is provided in Table 3 *.*

**TABLE 3:** Summary of all performance metrics of baseline, midterm and final assessments. Individual P values were calculated using individual sample t-tests with Welch’s correction.

Baseline				
	Intervention group	Control group		
Metrics	Mean (SD)	Mean (SD)	P value	Cohen’s d
Time (s)	1,125 (294)	1,082 (336.4)	0.801	0.14
Combined instrument tip path length (m)	16.07 (4.09)	17.52 (4.76)	0.553	−0.33
Combined instrument angular path (deg)	3110 (898.8)	3338 (1,021)	0.665	−0.24
Needle dropped	19 (10.25)	23.43 (11.77)	0.468	−0.40
Number of failed stitch attempts	12.14 (8.34)	9.57 (5.32)	0.507	0.37
Combined instruments out of view	6.57 (5.88)	4.86 (1.47)	0.534	0.37

Midterm				
	Intervention group	Control group		
Metrics	Mean (SD)	Mean (SD)	P value	Cohen’s d
Time (s)	863.6 (349.8)	565.4 (294.1)	0.111	0.90
Combined instrument tip path length (m)	12.7 (4.1)	9.9 (5.5)	0.311	0.56
Combined instrument angular path (deg)	2,209 (617.1)	1828 (929.5)	0.386	0.47
Needle dropped	13.4 (6.9)	9.4 (3.4)	0.204	0.74
Number of failed stitch attempts	12.7 (11.3)	7.6 (6.7)	0.325	0.53
Combined instruments out of view	3.3 (4.6)	1.4 (1.7)	0.345	0.53

Final assessment				
	Intervention group	Control group		
Metrics	Mean (SD)	Mean (SD)	P value	Cohen’s d
Time (s)	526.6 (193.4)	404.3 (93.94)	0.168	0.76
Combined instrument tip path length (m)	6.95 (1.31)	6.32 (1.59)	0.432	0.43
Combined instrument angular path (deg)	1,266 (239.4)	1,175 (379.9)	0.603	0.27
Needle dropped	3.86 (1.57)	2.43 (1.90)	0.153	0.81
Number of failed stitch attempts	3.14 (2.34)	4.86 (4.95)	0.430	−0.45
Combined instruments out of view	1.14 (1.22)	1 (1.155)	0.825	0.12

## Crossover trial

**FIGURE 3:**
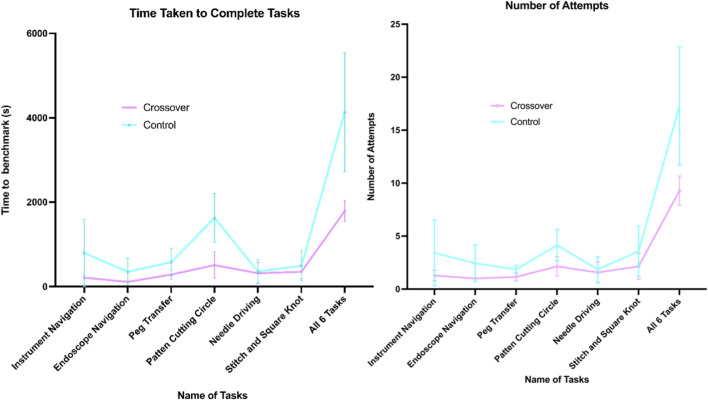
Graphs of time (s) and number of attempts taken to complete 6 FRS tasks.

A comparison of the time taken to complete six tasks on the VT between the control and crossover groups indicated a statistically significant difference in the total duration required for task completion. The control group had a mean total time of 69 min to achieve the benchmark across all tasks, while the crossover group averaged 30 min (mean difference of 39 min ±9.01 min, p = 0.004). Additionally, the mean number of attempts necessary to reach the benchmark was 17.3 for the control group and 9.3 for the crossover group (mean difference of 8 attempts ±2.2 attempts, p = 0.009). When analyzing each task individually, statistical significance was found only in the time taken to reach the benchmark for the Pattern Cutting Circle task (p = 0.001). Nonetheless, the crossover group consistently required less time and fewer attempts across all tasks compared to the control group (Figure 3)*.*

## Discussion

To our knowledge, this is the first study to directly compare a commercially available VR headset with a robotic surgery console for RAS training. As a feasibility study, our aim was to gain an initial understanding of the effectiveness of headset-based VR in developing RAS skills and to explore its potential as a supplementary training tool, particularly in resource-constrained settings. It is important to acknowledge that the present work was not designed or powered to provide conclusive evidence of equivalence between training modalities. With only 14 participants, variation between individuals had a marked influence on outcomes, and the confidence intervals around group differences were wide. As such, the findings should be regarded as an early step in exploring the role of headset-based VR, offering hypotheses and directions for subsequent, adequately powered investigations.

Within these constraints, both VT-VR and VT groups in our study showed improvement in RAS performance. While the absence of a placebo group limits internal comparison, prior research consistently demonstrates that console-based VR training outperforms no training. This suggests the observed improvements stem from the training interventions rather than task familiarity (Kawashima et al., 2024; Cho et al., 2013; Chien et al., 2013; Lendvay et al., 2013; Vaccaro et al., 2013; Raison et al., 2020). There were no significant differences between groups in midterm and final scores, task duration, or kinematic metrics. However, we could not conclusively establish non-inferiority. Although the mean difference of 8.4 in final composite scores fell within our predefined margin, the large standard error prevented definitive conclusions. Notably, the control group, more accustomed to the console’s controls and spatial interface, may have had an initial advantage. However, by the final assessment, the VT-VR group performed comparably. This finding may reflect a more rapid learning curve for console-based training—an observation warranting further investigation.

Furthermore, following the interventions, both groups completed a 10-point Likert scale rating assessing their comfort in manipulating robotic arms. The VT-VR group reported comfort levels comparable to those of the VT group (8.00 [SD, 1.29] vs. 8.14 [SD, 1.21]), with no statistically significant difference between cohorts (t = −0.21; P = 0.83). These findings indicate similar user experience between platforms and support the potential translational applicability of VT-VR training to console-based robotic systems.

The VR group also required, on average, 39 min less and eight fewer attempts to reach task benchmarks after crossing over to the console. While familiarity may partly explain this, RAS skill acquisition through the VR and the nature of VR controls likely contributed. The free-moving handsets amplify physiological tremors, increasing path length and occasionally pushing performance just outside threshold limits. Thus, participants needed to refine their precision and efficiency to meet benchmarks compared with the control group.

Compared to console-based training, the VT-VR has certain limitations. The controls are free-moving and lack a fixed axis, which makes it difficult for trainees to accurately grasp and replicate the necessary hand movements, such as the correct axis of pronation and supination, to control the robotic arms effectively. Moreover, while VT-VR offers immersive 3D perspectives, fine-detail visibility, such as needle grasping, can be difficult, and extended use often causes eye strain. In contrast, the Versius console’s high-resolution display allows for longer, more comfortable sessions.

Despite these shortcomings, the VT-VR offer distinct advantages, particularly in terms of cost and accessibility. The VIVE 3 headset, priced at around £1,372, is significantly cheaper than a complete surgical system like Versius, which costs £1.2 million. This affordability theoretically allows institutions to purchase multiple units for use in medical schools or hospitals and even enables at-home practice, negating travel time and coordinating access to the robot console during working hours. The portability of VT-VR, which can fit into a backpack, contrasts with the large and expensive console setups. Novice trainees could therefore first use the VR headset as an initial preparatory phase, particularly at home, to build foundational skills. This would then allow them to move on to the professional VR console for more refined and advanced practice. By making training more widely accessible, VR headsets have the potential to empower emerging surgeons and bridge gaps in training caused by limited access to consoles. VR headsets also have the potential to scale robotic training and may have a future role in aspects such as competitive access to robotic training posts, appraisal and credentialing, which would replicate the approach seen in aviation (Collins and Wisz, 2020).

The limitations of this study must also be recognised. The sample size of this study (n = 14) was extremely small, increasing the chance of type II errors. This, combined with the fact that the overall score calculated by the simulation was in increments of 20, resulted in a wide confidence interval. Furthermore, the inferiority margin was derived from a single validation study, which concluded that the increment of 20 was sufficient in determining an expert from a novice. While we believe the rationale is sufficiently conservative in preventing false conclusions of non-inferiority of the intervention to the control, the absence of precedent highlights the need for future studies to refine margin selection, ideally incorporating Delphi-style expert consensus or anchor-based approaches.

We acknowledge that the inclusion of medical students rather than surgical trainees limits the external validity of our findings in a clinical training context. Robotic surgery requires integration of technical skills with domain-specific procedural knowledge, situational awareness, and operative judgment that novices do not possess. Our study was therefore designed to isolate and evaluate the acquisition of basic robotic manipulation skills—a necessary but not sufficient component of surgical training. While this approach allowed us to control for prior robotic experience and focus on early psychomotor skill acquisition, future work should examine whether these findings translate to surgical trainees and practicing clinicians, where context-specific expertise plays a critical role.

Another important limitation is the platform-specific focus of this study. Given that the da Vinci system remains the predominant platform for robotic surgery worldwide, the translational relevance of this study within a clinical context is inherently constrained. The Versius console differs substantially from the more widely adopted da Vinci system (Intuitive, United States). Whereas da Vinci hand controls employ a pincer-like grip, with the thumb and middle finger positioned around the instrument grip and the index finger controlling the clutch, the Versius console adopts a configuration closer to a game controller: a trigger operated by the index finger controls the instrument jaws, thumbsticks provide camera control, and thumb-operated buttons manage clutch and energy functions. This design more closely mirrors the handheld controllers of VT-VR used in this study, likely facilitating more direct skill transfer from VR to console. While this similarity supports the face validity of our within-platform findings, it also constrains the broader translational relevance—our results may not generalize to platforms such as da Vinci, where controller ergonomics and haptic feedback differ.

However, with the advent of technologies such as the Apple Vision Pro—allowing for the manipulation of simulations without the need for handheld consoles, future investigations may be better positioned to examine robotic surgical systems that operate independently of gamified hand controllers, allowing for multi-system validations. Furthermore, following this feasibility study, future studies should focus on increasing the power of the study, have longer follow-up periods to assess retention of RAS skill, and include surgical trainees as well as conducting assessments in dry or wet models in order to improve external validity.

## Conclusion

Both VT-VR and VT training significantly improved fundamental RAS skills in novice participants over three training sessions. No statistically significant differences were observed between the two groups in composite scores for final and midterm assessments, nor in granular metrics such as time and kinematics, although a large effect size favouring the control group was found at the midterm assessment. Unfortunately, no definitive conclusions could be made regarding the non-inferiority of headset-based training over console-based RAS training given the small sample size and large confidence interval. Nevertheless, initiating training with the VR headset significantly decreased the time and number of attempts required to achieve benchmarks in FRS tasks on the surgical console. This study is constrained by limited sample size and external validity concerns due to sampling and compatibility of VIVE Focus hand-controllers and the Versius console, while the majority of robotic surgical systems continue to employ the da Vinci platform (Intuitive).

## Data Availability

The original contributions presented in the study are included in the article/Supplementary Material, further inquiries can be directed to the corresponding author.

## References

[B1] Althunian T. A. de Boer A. Groenwold R. H. H. Klungel O. H. (2017). Defining the noninferiority margin and analysing noninferiority: an overview. Br. J. Clin. Pharmacol. 83 (8), 1636–1642. 10.1111/bcp.13280 28252213 PMC5510081

[B2] Beulens A. J. W. Hashish Y. A. F. Brinkman W. M. Umari P. Puliatti S. Koldewijn E. L. (2021). Training novice robot surgeons: proctoring provides same results as simulator-generated guidance. J. Robotic Surgery 15 (3), 397–428. 10.1007/s11701-020-01118-y 32651769

[B3] Bjerrum F. Collins J. W. Butterworth J. Slack M. Konge L. (2023). Competency assessment for the versius surgical robot: a validity investigation study of a virtual reality simulator-based test. Surg. Endosc. 37 (10), 7464–7471. 10.1007/s00464-023-10221-8 37400688

[B4] Chien J. H. Suh I. H. Park S. H. Mukherjee M. Oleynikov D. Siu K. C. (2013). Enhancing fundamental robot-assisted surgical proficiency by using a portable virtual simulator. Surg. Innov. 20, 198–203. 10.1177/1553350612458545 22956399

[B5] Cho J. S. Hahn K. Y. Kwak J. M. Kim J. Baek S. J. Shin J. W. (2013). Virtual reality training improves da vinci performance: a prospective trial. J. Laparoendosc. Adv. Surg. Tech. 23, 992–998. 10.1089/lap.2012.0396 24138400

[B6] Cinelli M. Spada M. Kim W. Zhang Y. Burgherr P. (2021). MCDA index tool: an interactive software to develop indices and rankings. Environ. Syst. Decis. 41 (1), 82–109. 10.1007/s10669-020-09784-x 32837823 PMC7365520

[B7] Collins J. W. Wisz P. (2020). Training in robotic surgery, replicating the airline industry. How far have we come? World J. Urology 38 (7), 1645–1651. 10.1007/s00345-019-02976-4 31624867 PMC7303079

[B8] Farivar B. S. Flannagan M. Leitman I. M. (2015). General surgery residents' perception of robot-assisted procedures during surgical training. J. Surg. Educ. 72 (2), 235–242. 10.1016/j.jsurg.2014.09.008 25451717

[B9] Gurusamy K. S. Aggarwal R. Palanivelu L. Davidson B. R. (2009). Virtual reality training for surgical trainees in laparoscopic surgery. Cochrane Database Syst. Rev. (1), Cd006575. 10.1002/14651858.CD006575.pub2 19160288

[B10] Halvorsen F. H. Elle O. J. Dalinin V. V. Mørk B. E. Sørhus V. Røtnes J. S. (2006). Virtual reality simulator training equals mechanical robotic training in improving robot-assisted basic suturing skills. Surg. Endosc. Other Interventional Tech. 20, 1565–1569. 10.1007/s00464-004-9270-6 16902750

[B11] Kawashima K. Nader F. Collins J. W. Esmaeili A. (2024). Virtual reality simulations in robotic surgery training: a systematic review and meta-analysis. J. Robot. Surg. 19 (1), 29. 10.1007/s11701-024-02187-z 39688774

[B12] Khan Mustafa Tamim A. Patnaik R. Lee C. S. Willson C. M. Demario V. K. Krell R. W. (2023). Systematic review of academic robotic surgery curricula. J. Robotic Surg. 17 (3), 719–743. 10.1007/s11701-022-01500-y 36413255

[B13] Laver K. E. Lange B. George S. Deutsch J. E. Saposnik G. Crotty M. (2017). Virtual reality for stroke rehabilitation. Cochrane Database Syst. Rev. 11 (11), Cd008349. 10.1002/14651858.CD008349.pub4 29156493 PMC6485957

[B14] Lendvay T. S. Br C. T. White L. Kowalewski T. Jonnadula S. Mercer L. D. (2013). Virtual reality robotic surgery warm-up improves task performance in a dry laboratory environment: a prospective randomized controlled study. J. Am. Coll. Surg. 216, 1181–1192. 10.1016/j.jamcollsurg.2013.02.012 23583618 PMC4082669

[B15] Matteo Mazziotta A. P. (2013). Methods for constructing composite indices: one for all or all for one?. RIEDS - Rivista Italiana di Econ. Demografia e Statistica - Italian J. Econ. Demogr. Stat. Stud. 67, 67–80.

[B16] Raison N. Gavazzi A. Abe T. Ahmed K. Dasgupta P. (2020). Virtually competent: a comparative analysis of virtual reality and dry-lab robotic simulation training. J. Urology 199, e1137. 10.1089/end.2019.0541 31973576

[B17] Schumi J. Wittes J. T. (2011). Through the looking glass: understanding non-inferiority. Trials 12 (1), 106. 10.1186/1745-6215-12-106 21539749 PMC3113981

[B18] Song M. K. Lin F. C. Ward S. E. Fine J. P. (2013). Composite variables: when and how. Nurs. Res. 62 (1), 45–49. 10.1097/NNR.0b013e3182741948 23114795 PMC5459482

[B19] Tursø-Finnich T. Jensen R. O. Jensen L. X. Konge L. Thinggaard E. (2023). Virtual reality head-mounted displays in medical education: a systematic review. Simul. Healthc. 18 (1), 42–50. 10.1097/sih.0000000000000636 35136005

[B20] Vaccaro C. M. Crisp C. C. Fellner A. N. Jackson C. Kleeman S. D. Pavelka J. (2013). Robotic virtual reality simulation plus standard robotic orientation *versus* standard robotic orientation alone: a randomized controlled trial. Female Pelvic Med. Reconstr. Surg. 19, 266–270. 10.1097/spv.0b013e3182a09101 23982574

[B21] van der Kruk S. R. Zielinski R. MacDougall H. Hughes-Barton D. Gunn K. M. (2022). Virtual reality as a patient education tool in healthcare: a scoping review. Patient Educ. Couns. 105 (7), 1928–1942. 10.1016/j.pec.2022.02.005 35168856

[B22] van der Leun J. A. Siem G. Meijer R. P. Brinkman W. M. (2022). Improving robotic skills by video review. J. Endourology/Endourological Soc. 36 (8), 1126–1135. 10.1089/end.2021.0740 35262417

[B23] Vassantachart A. Y. Yeo E. Chau B. (2022). Virtual and augmented reality-based treatments for phantom limb pain: a systematic review. Innov. Clin. Neurosci. 19 (10-12), 48–57. 36591552 PMC9776775

[B24] Vigran H. J. Diaz S. Suryadevara A. Blair Y. Aubry S. Reddy R. M. (2024/10/13 2024). Assessing the need for robotic surgery training in standard medical education: insights from medical students. Discov. Educ. 3 (1), 176. 10.1007/s44217-024-00284-7

