# Supplementary material for: A feasibility study: a non-inferiority study comparing head-mounted and console-based virtual reality for robotic surgery training
Source: Front Robot AI. 2026 Jan 6;12:1616462. doi: 10.3389/frobt.2025.1616462 (PMC12816980; doi:10.3389/frobt.2025.1616462)

Appendix 1: Images of the Versius Trainer (VT) and Versius Trainer in Virtual Reality (VT-VR)

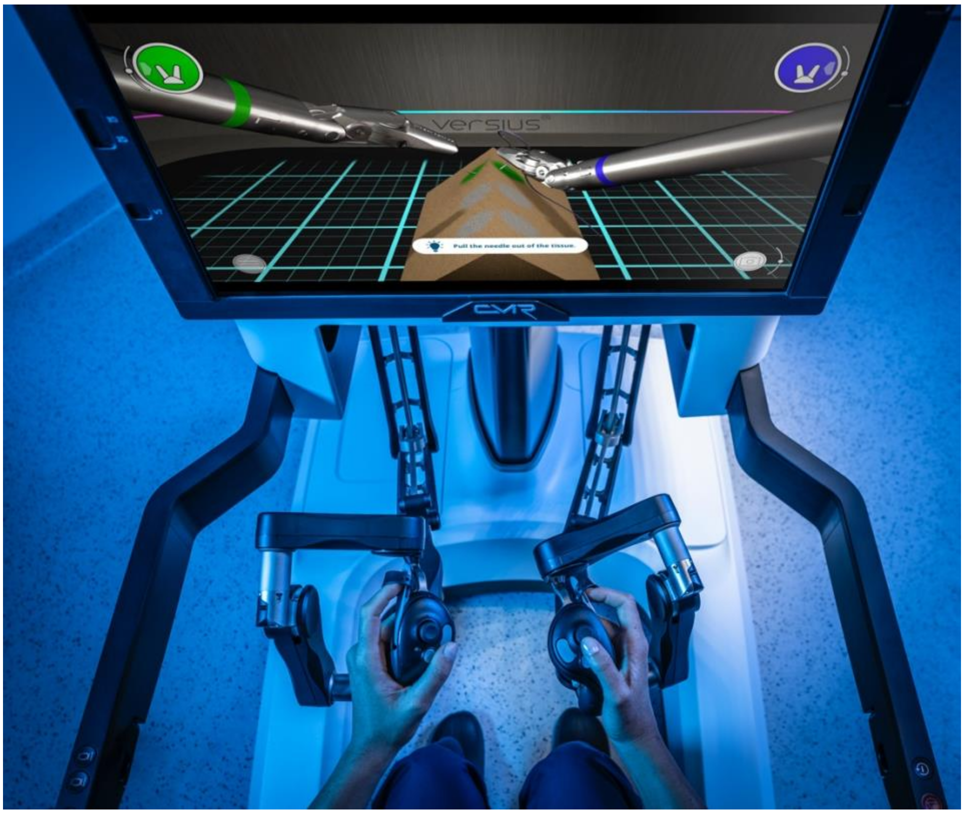

Versius Trainer (VT) (Photo used with permission from CMR Surgical)

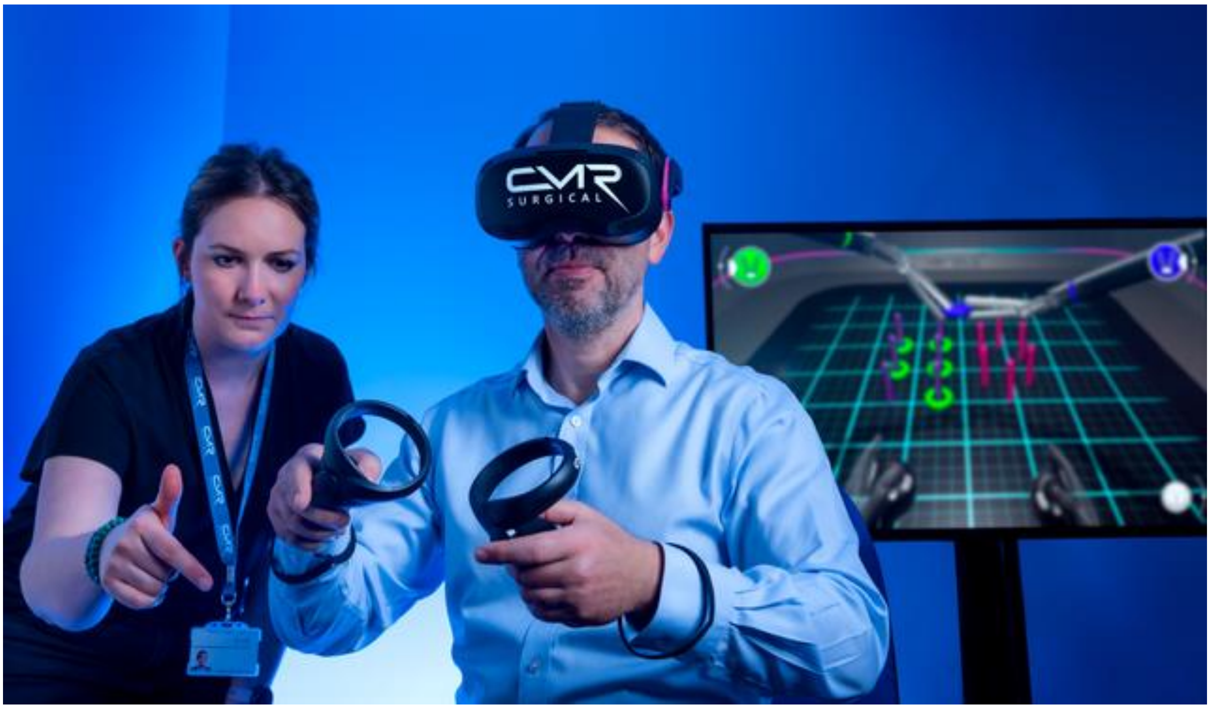

Versius Trainer in Virtual Reality (Photo used with permission from CMR Surgical)

Appendix 2: Learning Points of the FRS Tasks and Sample Calculation of Overall Score of the Assessment Task

A. **Instrument Navigation:** Participants are required to move two robotic arms to the center of 3-dimensional balls that appear on the screen. This task teaches the mechanics of clutching, moving instruments efficiently, keeping instruments in view, and to moving both arms simultaneously.

B. **Endoscope Control:** Participants must operate the endoscope, mastering the three axes of camera movement: panning, tilting, and zooming. This task aims to develop proficiency in handling the endoscope with precision.

C. **Peg Transfer:** This task involves transferring rings from one peg to another by passing the ring between the two robotic arms. It aims to enhance dexterity in using robotic arms, and precision in holding objects at the correct angle and at the instrument's tip.

D. **Pattern Cutting Circle:** Participants are required to cut along two lines forming a circle. This task is designed to improve precise movements of the robotic arms whilst using the endowrists, which allows instruments to tilt in different angles.

E. **Needle Driving: This** task involves passing a needle through three specified areas on a suture sponge. It aims to teach suturing mechanics, including holding the needle at the tip in a perpendicular fashion, performing needle passage via supination and pronation while using clutches, and transferring the needle between hands.

F. **Stitch and Square Knot:** Participants are instructed to pass a stitch through a suture sponge and tie two square knots. This task focuses on teaching the techniques of pulling sutures through (via walking the suture) and tying a square knot correctly.

G. **Running Suture:** Participants are tasked with stitching, tying two square knots, and placing three consecutive running sutures through a suture sponge. This task requies the integration of all previously learned skills in order to be completed sucessfully.

Task	Kinematic Metrics	Sample Result	Threshold	Pass/Fail	Overall Score
Running suture	Combined instrument tip path length (m)	6.5	7.14	Pass	**80%** *Did not reach benchmark*
	Combined instrument angular path (deg)	1240	1340	Pass	
	Needle dropped	1	3	Pass	
	Number of failed stitch attempt	1	6	Pass	
	Combined instruments out of view	15	14	Fail	

Appendix 3: Images of the FRS Simulation Tasks

Instrument Navigation

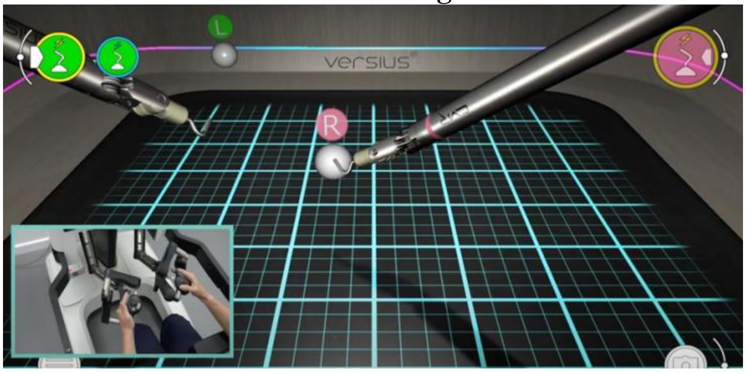

Endoscope Control

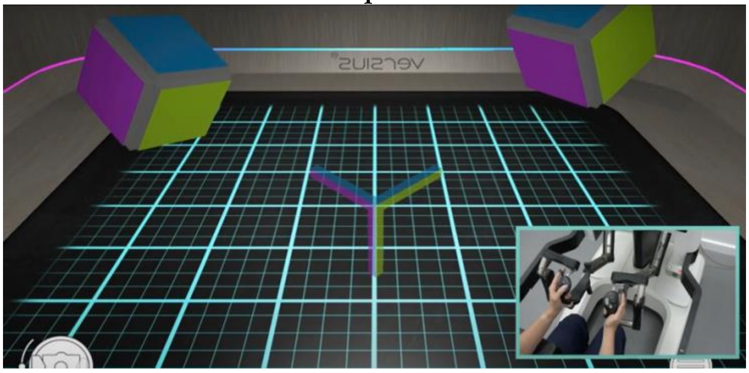

Peg Transfer

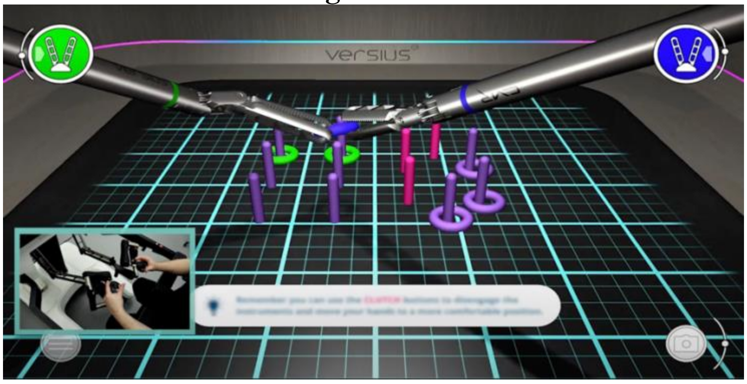

Pattern Cutting Circle

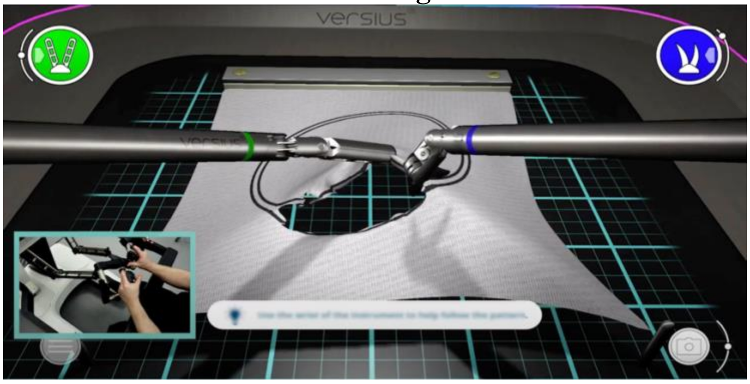

Needle Driving

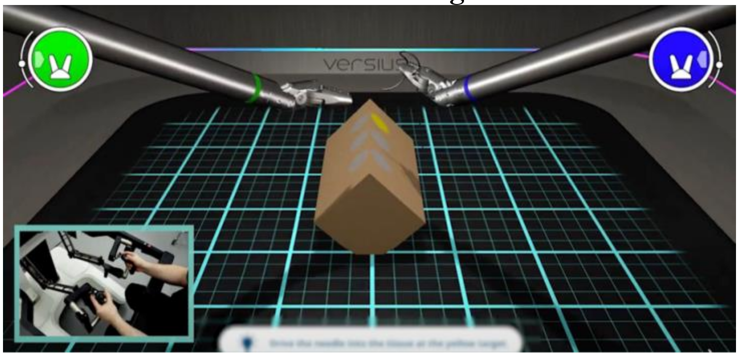

Stitch and Square Knot

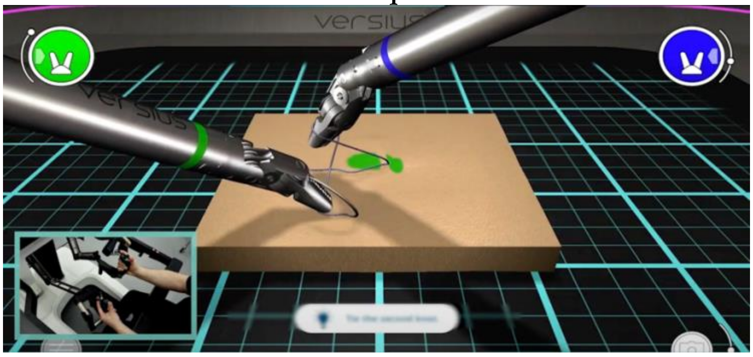

Running Suture

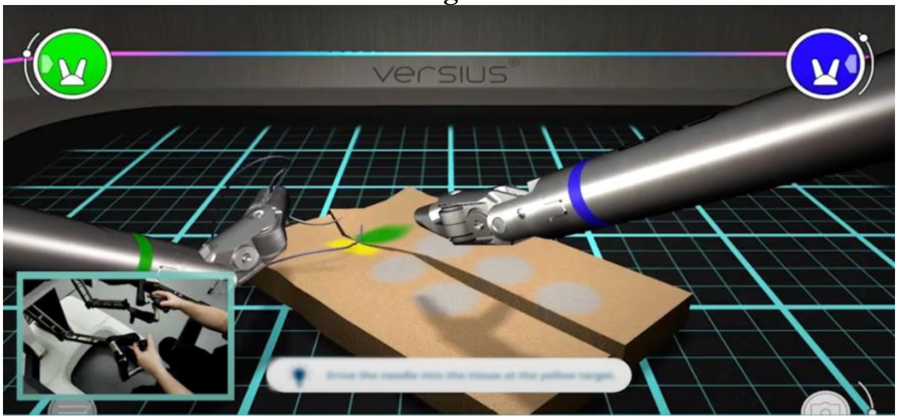

Supplement: Supplementary file 1 [file DataSheet1.pdf]
